# Long non-coding RNA TUG1 enhances chemosensitivity in non-small cell lung cancer by impairing microRNA-221-dependent PTEN inhibition

**DOI:** 10.18632/aging.102271

**Published:** 2019-09-18

**Authors:** Shenghu Guo, Lei Zhang, Yuehua Zhang, Zheng Wu, Dongwei He, Xing Li, Zhiyu Wang

**Affiliations:** 1 Department of Immuno-Oncology, The Fourth Hospital of Hebei Medical University, Shijiazhuang, Hebei 050011, P. R. China

**Keywords:** non-small cell lung cancer, TUG1, autophagy, chemotherapy, PTEN

## Abstract

Long non-coding RNA taurine up-regulated gene 1 (TUG1) emerges as new players in gene regulation in several cancers; however, its mechanism of action in non-small cell lung cancer (NSCLC) has not been well-studied. Herein, we determined expression pattern of TUG1 in NSCLC and further identified its effect on the chemosensitivity of NSCLC. Low expression of TUG1 was found in NSCLC tissues obtained from non-responders to platinum-based chemotherapy and reflected poor overall survival. TUG1 overexpression was shown to inhibit cell proliferation, migration, invasion, but facilitate apoptosis and autophagy in NSCLC cells resistant to cisplatin (DDP). Smaller size of tumor xenografts of DDP resistant NSCLC cells in the presence of TUG1 demonstrated enhancement of chemosensitivity by TUG1 *in vivo*. High expression of miR-221 and low expression of PTEN were determined in cancer tissues obtained from non-responders to platinum-based chemotherapy and reflected poor overall survival. TUG1 inhibited miR-221 that targeted PTEN, as evidenced by an elevated expression of PTEN in the presence of miR-221 or the absence of TUG1. Our present study reveals a model of enhancement of chemosensitivity that consists of TUG1, miR-221 and PTEN. Modulation of their levels may offer a new approach for improving anti-tumor efficacy for chemotherapeutic agents in NSCLC.

## INTRODUCTION

Lung cancer is the leading cause of cancer-related death worldwide. In 2012 approximately 1.6 million of people died of lung cancer, and the number of deaths will increase to 3 million by 2035 globally [1]. Lung cancer ranked first as the leading causes of cancer death among Chinese population in 2015 [2]. Human cancers occur in elderly population at an alarming rate, indicating age is the greatest risk factor for cancer. This demographic shift is the leading reason for an increase of cancer incidence. Lung cancer is a prevalent disease occurring in the elderly patients [3]. Non-small cell lung cancer (NSCLC) is the most common type and accounts for 85% of all lung cancer cases [4]. Despite advances in developing non-cytotoxic targeted agents, chemotherapy still remains the mainstay in treating advanced lung cancer. However, resistance to chemotherapy, either intrinsic or acquired, is the leading impediment that limits the continued effective treatment [5]. Recently, there has been a growing interest in seeking epigenetic mechanisms of drug resistance in lung cancer [6], including long non-coding RNAs (lncRNAs) [7] and microRNAs (miRNAs) [8].

LncRNAs represent a diverse class of transcripts that are emerging as new players in regulating gene transcription and governing biological processes [9]. LncRNA taurine-upregulated gene 1 (TUG1), originally identified as a transcript up-regulated by taurine, is expressed in a tissue specific pattern and acts as either an oncogene or a tumor suppressor in human cancers [10]. For instance, TUG1 has been reported as a tumor suppressor in human glioma, while it highlighted a potential oncogenic role in osteosarcoma [11, 12]. Expression patterns of TUG1 were investigated in several lung cancer studies [13, 14], while there are conflicting data with respect to the role of TUG1 in lung cancer. Current insights into the molecular systems of lncRNA-miRNA-mRNA regulatory interactions and implications in lung cancer allow a hypothesis that TUG1 may be involved in the regulatory network of mRNA via ceRNA mediated miRNA evasion [15]. A previous study reported targeted inhibition of phosphatase and tensin homologue deleted on chromosome 10 (PTEN) by miRNA-221 and their associations with drug resistance in lung cancer [16]. In this study, we attempted to explore the interactions among TUG1, miRNA-221 and PTEN in NSCLC and whether the TUG1/miR-221/PTEN axis regulates chemosensitivity. We found lower expression of TUG1 in cancer tissues obtained non-responders to platinum-based chemotherapy in contrast to responders and reflected poor overall survival. Subsequently, we prepared a pcDNA3.1-based vector containing TUG1 and found that TUG1 overexpression impaired cell proliferation, migration, invasion, but facilitated apoptosis and autophagy in NSCLC cells resistant to DDP. Additionally, we showed that TUG1 upregulated PTEN *via* miR-221, thus inhibiting NSCLC cells. Finally, these *in vitro* findings were reproduced in tumor xenografts of DDP resistant NSCLC cells using BALB/c mice following DDP treatment.

## RESULTS

### Baseline characteristics of included participants

**Figure 1:**
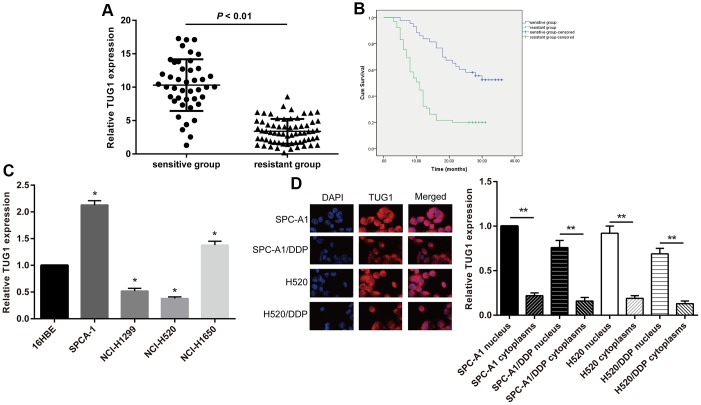
**The TUG1 expression level and the intracellular localization.** (**A**) The TUG1 expression level of NSCLC patients in the resistant and sensitive groups; (**B**) survival conditions of NSCLC patients in the resistant and sensitive groups; (**C**) expression level of TUG1 determined by qRT-PCR in NSCLC cells; (**D**) the intracellular localization by fluorescence *in situ* hybridization in NSCLC cells. ** *p* < 0.01.

To assess the effect of lncRNA TUG1 on chemoresistance in NSCLC, we classified patients into resistant and sensitive groups based on their response to chemotherapy. Based on the clinical data of the two groups (Table 1), we found no significant differences in gender and age between the sensitive and resistant groups. However, more patients with smoking history, poor differentiation, lymph node metastasis, and II-IV TNM staging were observed in the resistant group than the sensitive group (*p* < 0.05). According to the qRT-PCR detection of TUG1 expression level in each patient (Figure 1A), the TUG1 expression of the patients in the resistant group was significantly lower than that in the sensitive group (*p* < 0.01). All patients were followed up for a median follow-up of 14 months. Kaplan-Meier method was used to analyze the overall survival of the NSCLC patients. The total survival time of the sensitive group (26.93 ± 1.63 months) was significantly higher than that of the resistant group (13.48 ± 1.17 months) (Figure 1B).

**Table 1:** The clinical data of NSCLC patients in the resistant and sensitive groups.

**Item**	**Sensitive group (n = 43)**	**Resistant group (n = 65)**	***p* value**
Gender			0.695
Male	24	40	
Female	19	25	
Age (years)			0.879
< 60	26	39	
≥ 60	17	26	
Smoking history			0.014
Yes	12	35	
No	31	30	
Differentiation degree			< 0.001
Poor	12	46	
High/Medium	31	19	
Tumor node metatstasis			0.003
Yes	15	43	
No	28	22	
TNM staging			0.006
I	24	18	
II-IV	19	47	

### Expression level of TUG1and the intracellular localization in NSCLC cells

In this study, NSCLC cell lines SPC-A1, NCI-H1650, NCI-H520 and NCI-H1299 in addition to the normal epithelial cell line 16HBE of lung mucosa were selected. By comparing the expression of TUG1 in cells, we revealed that, SPC-A1 cells had the highest expression of TUG1 and NCI-H520 had the lowest relative to 16HBE cells (Figure 1C). So we selected these two cell lines for subsequent experiments. The effects lncRNAs exert is closely implicated in its cellular localization. LncRNAs located in the nucleus play a major role in transcriptional regulation, and lncRNAs located in the cytoplasm mainly play a role in post-transcriptional regulation. Therefore, we isolated the nucleus and cytoplasm, and observed the intracellular localization of TUG1 by fluorescence *in situ* hybridization. Fluorescence *in situ* hybridization showed that TUG1 was mainly localized in the nucleus, and a small amount was localized in the cytoplasm. The fluorescence intensity of TUG1 in drug-resistant cells was significantly weaker than that of the parental cells (Figure 1D).

### Overexpressed TUG1 promotes sensitivity of NSCLC cells to DDP

**Figure 2:**
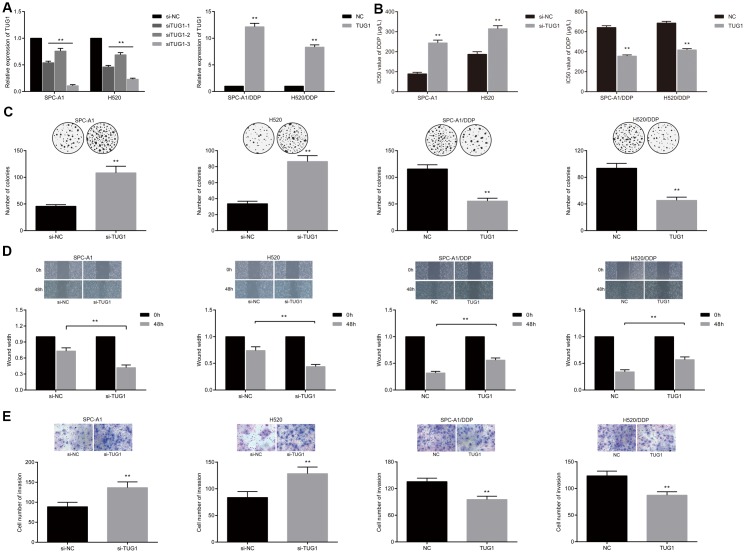
**Overexpression of TUG1 enhanced the sensitivity of NSCLC cells to DDP.** (**A**) the expression level of TUG1 after transfection of pcDNA-TUG1 and si-TUG1 detected by qRT-PCR; (**B**) the IC50 values of DDP in SPC-A1 cells and H520 cells transfected with si-TUG1 and SPC-A1/DDP and H520/DDP cells transfected with pcDNA-TUG1; (**C**) colony forming ability of transfected cells in each group under DDP treatment detected by colony formation assay; (**D**) migration ability of transfected cells in each group under DDP treatment detected by scratch test; (**E**) the invasive ability of transfected cells in each group detected by Transwell assay; ** *p* < 0.01 versus the si-NC group.

To investigate the possible effects of lncRNA TUG1 on chemoresistance in NSCLC, SPC-A1 and H520 cells were transfected with si-TUG1 and si-NC, respectively, besides, the SPC-A1/DDP and H520/DDP cells were transfected with pcDNA-TUG1 and pcDNA3.1. The transfection efficiency of TUG1 was detected by qRT-PCR. Compared with the si-NC group, siRNAs significantly down-regulated the expression of TUG1 in SPC-A1 and H520 cell lines, with the efficiency of siTUG1-3 being the most significant. The expression of TUG1 in SPC-A1/DDP and H520/DDP cells transfected with pcDNA-TUG1 was significantly up-regulated (Figure 2A). The effect of TUG1 on the IC50 value of DDP-induced NSCLC cell line was detected by MTT assay. The IC50 values of DDP in SPC-A1/si-TUG1 or H520/si-TUG1 cells were significantly elevated, versus the si-NC group (Figure 2B, *p* < 0.01). Conversely, IC50 values of DDP in SPC-A1/DDP/TUG1 or H520/DDP/TUG1 cells were lower than those in the si-NC group (Figure 2B, *p* < 0.01). The results indicated that TUG1 enhanced the sensitivity to DDP in NSCLC. Through colony formation experiments, we obtained similar results. When exposed to DDP, the SPC-A1/si-TUG1 cells and H520/si-TUG1 cells displayed ability to significantly enhanced ability of forming colonies after overactivation of TUG1. Whereas, the overexpression of TUG1 reduced the ability of TUG1-SPC-A1/DDP cells and H520/DDP cells to form colonies (Figure 2C). Through scratch test and Transwell assay, we demonstrated that cell migration and invasion were enhanced after inactivation of TUG1 in SPC-A1/si-TUG1 cells and H520/si-TUG1 cells, whereas SPC-A1/DDP cells and H520/DDP cells overexpressing TUG1 showed diminished migration and invasion ability (Figure 2D, 2E).

### Overexpressed TUG1 promotes autophagy, apoptosis and senescence of NSCLC cells

**Figure 3:**
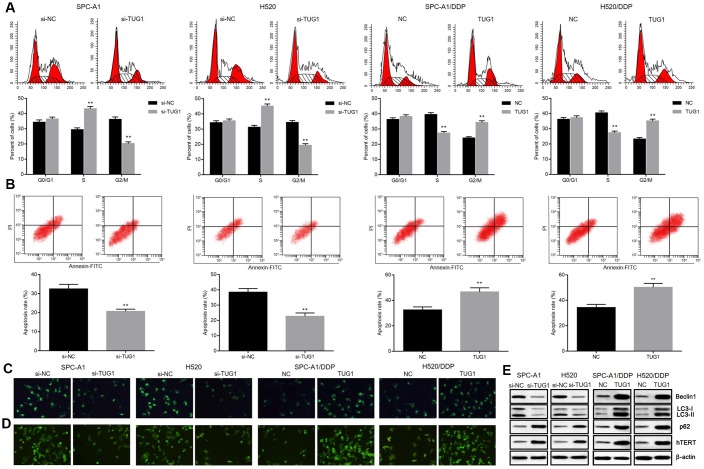
**Overexpression of TUG1 enhanced the apoptosis, autophagy and senescence of NSCLC cells in response to DDP.** (**A**) the effect of TUG1 on the cell cycle distribution of NSCLC cells; (**B**) the effect of TUG1 on apoptosis of NSCLC cells; (**C**) GFP-LC fusion protein tagging detection of autophagosome formation; (**D**) MDC staining to detect formation of autophagic vacuoles; (**E**) expression of autophagy-related proteins and cell senescence-related protein hTERT determined by Western blot analysis, normalized to β-actin. ** *p* < 0.01 versus the control group.

To further demonstrate the mechanism by which TUG1 inhibits NSCLC cell proliferation, we performed flow cytometry to assess cell cycle and apoptosis. As shown in Figure 3A, 3B, knockdown of TUG1 increased the percentage of NSCLC cells arrested in S phase and decreased the percentage of cells arrested in G2/M phase, compared to the NC group. In contrast, overexpression of TUG1 induced a decrease in the percentage of cells arrested in S phase in SPC-A1/DDP and H520/DDP cells, and an increase in the percentage of cells arrested in G2/M phase (*p* < 0.05). After exposure to DDP for 24 hours, SPC-A1/si-TUG1 or H520/si-TUG1 cells showed greater resistance to DDP-induced apoptosis, whereas SPC-A1/DDP/TUG1 or H520/DDP/TUG1 cells were more sensitive to DDP-induced apoptosis (*p* < 0.05). We used GFP-LC3 fusion protein tagging method and MDC staining to observe the autophagy of NSCLC cells. Distribution of GFP-LC3 protein in the cytoplasm indicated the absence of autophagy. The GFP-LC3 protein was localized to autophagosomes during autophagy. Each green spot indicated an autophagosome under the inverted fluorescence microscope, and the numbers of green spots were used to reflect the extent of autophagy. According to the fluorescence expression of GFP-LC3 fusion protein, after TUG1 was inactivated, SGC-A1 and H520 cells showed decreased fluorescence intensity as compared with the control group, indicating that autophagosome formation was decreased. However, after overexpression of TUG1, the fluorescence intensity in SPC-A1/DDP and H520/DDP cells increased, indicating an increase in autophagosome formation (*p* < 0.05, Figure 3C). Based on the results of MDC staining, MDC-positive cells in SPC-A1 and H520 cells were decreased after inactivation of TUG1, indicating that autophagic vacuole formation was suppressed. When TUG1 was overexpressed, the number of MDC-positive cells in the SPC-A1/DDP and H520/DDP cells increased, indicating an increase in autophagic vacuoles (*p* < 0.05, Figure 3D). By measuring the expression of autophagy-related proteins, we observed that the expression of Beclin1 and LC3II/I was decreased and the expression of p62 was increased in SPC-A1 and H520 cells after inactivation of TUG1. Whereas, the expression of Beclin1 and LC3II/I was increased and the expression of p62 was diminished in SPC-A1/DDP and H520/DDP cells after overexpressing TUG1 (*p* < 0.05, Figure 3E). In addition, we determined the expression of hTERT by Western blot analysis and found that the expression of hTERT was elevated in the absence of TUG1 and the expression was reduced in the presence of TUG1 in SPC-A1/DDP and H520/DDP cells (*p* < 0.05). These data indicate that TUG1 can enhance autophagy, apoptosis and senescence of NSCLC cells and increase chemosensitivity.

### Overexpressed TUG1 promotes sensitivity of xenograft tumors to DDP in vivo

**Figure 4:**
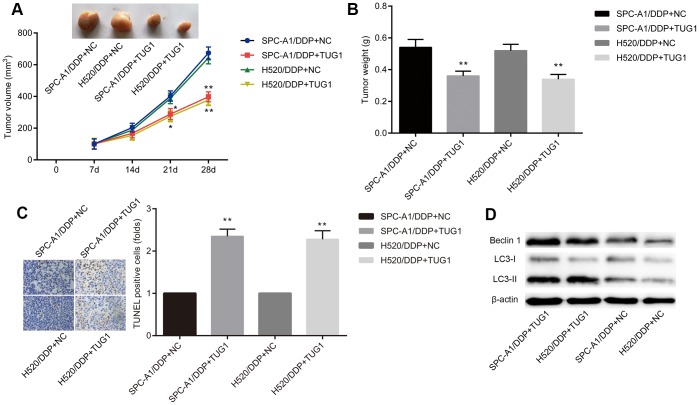
**Overexpression of TUG1 enhances chemosensitivity of NSCLC cells to DDP in nude mice.** (**A**) tumor volume changes in mice with NSCLC; (**B**) tumor weight in mice with NSCLC; (**C**) TUNEL staining of apoptosis in NSCLC mice (× 400); (**D**) the expression of autophagy-related proteins in NSCLC tissues in vivo. * *p* < 0.05 versus the NC group; ** *p* < 0.01.

To investigate the effect of TUG1 on NSCLC cells resistant to DDP in vivo, SPC-A1/DDP and H520/DDP cells stably transfected with TUG1 were transplanted into nude mice. Two weeks later, after intraperitoneal injection of 1 mg/kg DDP, all tumors were resected. By DDP treatment, the tumors formed by SPC-A1/DDP and H520/DDP cells transfected with TUG1 were smaller in size and lighter in weight than those in the NC groups (Figure 4A, 4B). TUNEL staining showed more apoptosis in tumors overexpressing TUG1 (Figure 4C). In addition, we performed Western blot assay to detect the expression of autophagy-related proteins Beclin1 and LC3 in subcutaneous tumors formed by cells transfected with TUG1. As shown in Figure 4D, the expression of Beclin1 and LC3II/I was increased in the SPC-A1/DDP and H520/DDP cells transfected with TUG1, compared with the NC groups (*p* < 0.05). These results indicate that overexpression of TUG1 sensitizes DDP-resistant NSCLC cells to DDP treatment and enhances autophagy and apoptosis.

### Overexpressed TUG1 promotes sensitivity of xenograft tumors to DDP *in vivo*

**Figure 5:**
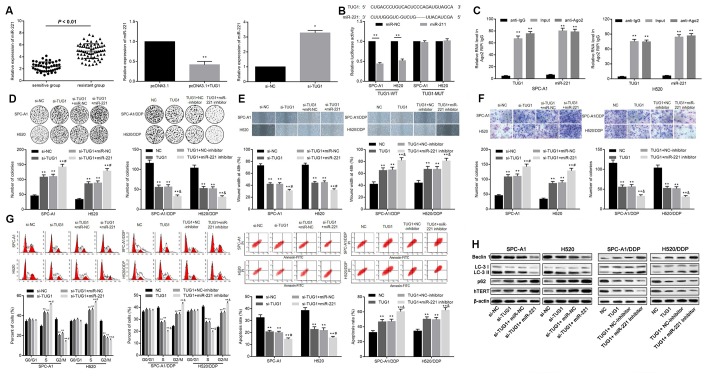
**Relationship between TUG1 and miR-221 and their effects on chemoresistance in NSCLC.** (**A**) the expression level of miR-221 in the sensitive group and the resistant group and the relationship with TUG1; (**B**) the dual luciferase reporter assay to verify the targeting relationship of TUG1 and miR-221; (**C**) RIP analysis with the Ago2 antibody, and then quantitative analysis of TUG1 and miR221 by qRT-PCR; (**D**) colony formation to detect the effect of TUG1 and miR-221 on cell colony formation ability; (**E**) scratch test to detect the effect of TUG1 and miR-221 on cell migration ability; (**F**) Transwell invasion assay to detect the effect of TUG1 and miR-221 on the cell invasive ability; (**G**) flow cytometry to detect the effects of TUG1 and miR-221 on cell cycle and apoptosis; (**H**) expression of autophagy-related proteins and cell senescence-related protein hTERT determined by Western blot analysis, normalized to β-actin. * *p* < 0.05 versus the control group; ** *p* < 0.01; # *p* < 0.05 versus the si-TUG1 group; & *p* < 0.05 versus the TUG1 group.

In order to determine whether TUG1 acts as a ceRNA of miR-221, we first examined the expression levels of miR-221 in the resistant group and the sensitive group, and found that the expression level of miR-221 in the sensitive group was significantly lower than that in the resistant group (*p* < 0.01). The expression level of miR-221 was increased when TUG1 was inactivated, and the level of miR-221 was decreased when TUG1 was overexpressed (*p* < 0.01, Figure 5A). To further validate the relationship between miR-221 and TUG1, dual luciferase reporter assay and RIP assay revealed a competing relationship between TUG1 and miR-221 (Figure 5B, 5C), suggesting that miR-221 is a direct target of TUG1. Based on colony formation experiments, in SPC-A1 and H520 cells, transfection of si-TUG1, si-TUG1 + miR-NC, and si-TUG1 + miR-221 resulted in enhanced ability of colony formation, cell migration and invasion. Besides, the ability of colony formation, cell migration and invasion of SPC-A1/DDP cells and H520/DDP cells transfected with TUG1, TUG1 + NC-inhibitor, TUG1 + miR-221 inhibitor was significantly reduced (*p* < 0.01, Figure 5D–5F). Relative to the NC group, in SPC-A1 and H520 cells, transfection of si-TUG1, si-TUG1 + miR-NC, si-TUG1 + miR-221 increased the percentage of cells in S phase and decreased that in G2/M phase, accompanied by decreased apoptosis. On the contrary, the overexpression of TUG1 induced the decrease of the percentage of cells in S phase in SPC-A1/DDP and H520/DDP cells, and the percentage of cells in G2/M phase increased, accompanied by promoted apoptosis (*p* < 0.01, Figure 5G). Western blot analysis was used to detect the expression of autophagy-related proteins and cell senescence-related protein hTERT. Compared with the N group, the expression of Beclin1 and LC3II/I was decreased and the expression of p62 and hTERT was elevated in SPC-A1 and H520 cells transfected with si-TUG1, si-TUG1 + miR-NC and si-TUG1 + miR-221. While transfection of TUG1, TUG1 + NC-inhibitor, TUG1 + miR-221 inhibitor resulted in increased expression of Beclin1 and LC3II/I as well as reduced expression of the expression of p62 and hTERT was in SPC-A1/DDP and H520/DDP cells (*p* < 0.05, Figure 5H).

### TUG1 increases the expression of PTEN, a target of miR-221.

**Figure 6:**
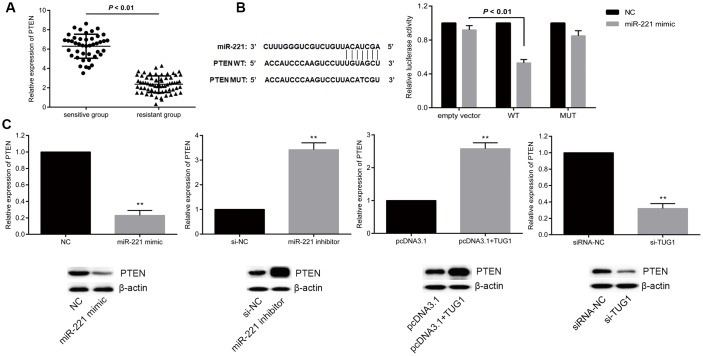
**miR-221 targets PTEN and PTEN is positively regulated by TUG1.** (**A**) PTEN expression level in the sensitive and resistant groups of NSCLC; (**B**) dual luciferase report assay to verify the targeting relationship between miR-221 and PTEN; (**C**) regulation of miR-221 and TUG1 to PTEN expression level. * *p* < 0.05 versus the NC group; ** *p* < 0.01.

PTEN is a well-recognized tumor suppressor by inhibiting the PI3K signaling pathway in several human cancers including lung cancer. PTEN has been shown to be the target of miR-221. In this case, we proposed a hypothesis that TUG1 could function as a ceRNA and increased the expression of PTEN. To end it, we examined the expression levels of PTEN in the resistant and sensitive groups and found that the expression level of PTEN in the sensitive group was significantly higher than that in the resistant group (*p* < 0.01, Figure 6A). To further verify the relationship between miR-221 and PTEN, dual luciferase reporter assay revealed that PTEN is a direct target of miR-221 (Figure 6B). Transfection of miR-221 mimic or si-TUG1 reduced the expression level of PTEN, while transfection of miR-221 inhibitor or pcDNA-TUG1 increased the expression level of PTEN (*p* < 0.01, Figure 6C), indicating that TUG1 positively regulated PTEN, and PTEN is a direct target of miR-221.

### Overexpressed TUG1 promotes sensitivity of NSCLC cells by positively regulating PTEN

**Figure 7:**
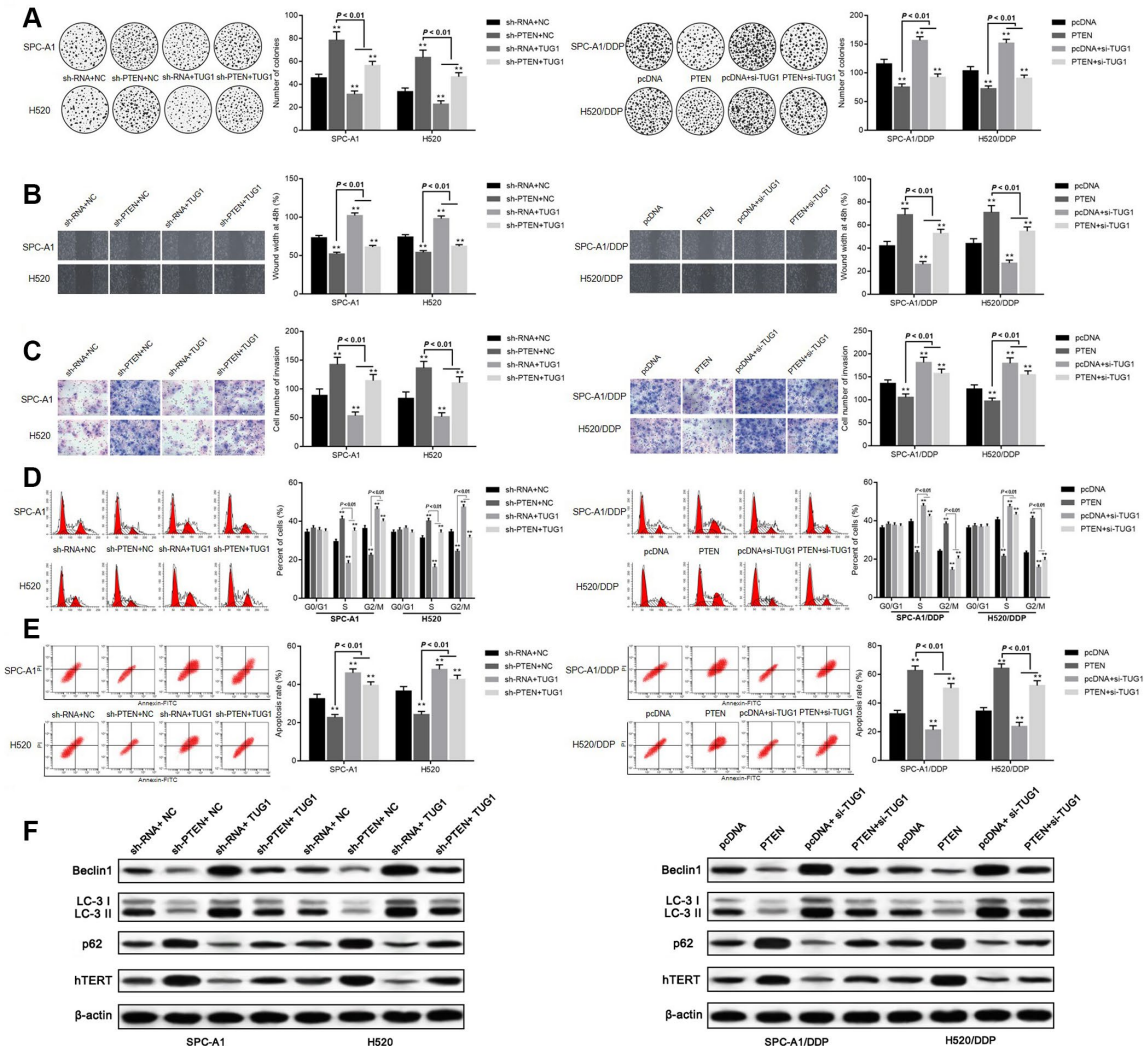
**Overexpression of TUG1 promotes sensitivity of NSCLC cells by positively regulating PTEN.** (**A**) colony formation assay to detect the effect of TUG1 and PTEN on cell colony formation ability; (**B**) scratch test to detect the effect of TUG1 and PTEN on cell migration ability; (**C**) Transwell invasion assay to detect the effect of TUG1 and PTEN on cell invasion ability; (**D**) flow cytometry to detect the effect of TUG1 and PTEN on cell cycle; (**E**) flow cytometry to detect the effect of TUG1 and PTEN on cell apoptosis; (**F**) expression of autophagy-related proteins and cell senescence-related protein hTERT determined by Western blot analysis, normalized to β-actin. * *p* < 0.05 versus the NC group; ** *p* < 0.01.

The effect of TUG1 on the influence of PTEN on chemoresistance in NSCLC was further validated. According to the colony formation assay, SPC-A1 and H520 cells transfected with sh-PTEN + NC and sh-PTEN + TUG1 manifested facilitated colony formation, migration and invasion ability. Transfection of sh-RNA + TUG1 reduced the ability of cells to form colonies, migrate and invade. Whereas SPC-A1/DDP cells and H520/DDP cells overexpressing PTEN, or transfected with PTEN + si-TUG1 demonstrated significantly reduced ability of colony formation, migration and invasion. The ability of colony formation, migration and invasion of SPC-A1/DDP cells and H520/DDP cells transfected with pcDNA+si-TUG1 was significantly enhanced (*p* < 0.05, Figure 7A–7C). Versus the NC group, transfection of sh-PTEN + NC and sh-PTEN + TUG1 in SPC-A1 and H520 cells increased the percentage of cells in the S phase, decreased the percentage of cells in the G2/M phase, corresponding to decreased apoptosis. On the contrary, overexpression of PTEN and transfection of PTEN + si-TUG1 induced a decrease in the percentage of cells in S phase and an increase in the percentage of cells in G2/M phase for SPC-A1/DDP and H520/DDP cells, in addition to more significant apoptosis (*p* < 0.05, Figure 7D, 7E). The expression of autophagy-related proteins and cell senescence-related protein hTERT was detected by Western blot assay. Compared with the NC groups, the expression of Beclin1 and LC3II/I was decreased and the expression of p62 and hTERT was elevated in SPC-A1 and H520 cells after transfection with sh-PTEN + NC and sh-PTEN + TUG1. After transfection of PTEN + si-TUG1, the expression of Beclin1 and LC3II/I was increased and the expression of p62 and hTERT was diminished in SPC-A1/DDP and H520/DDP cells (*p* < 0.05, Figure 7F).

## DISCUSSION

Recently, a review provided a comprehensive update on pharmacogenomics of platinum-based chemotherapy sensitivity in NSCLC [17], which underlines the role of molecular analysis in regulating chemotherapy sensitivity NSCLC. LncRNAs have emerged as a new paradigm for lung cancer [18]. We hypothesize that TUG1, miR-221 and PTEN constitute an axis in the machinery of response to chemotherapy in NSCLC cells. In detail, in contrast to responders to platinum-based chemotherapy, lower expression of TUG1 determined in cancer tissues obtained from non-responders may be associated with poor overall survival. TUG1 overexpression could inhibit NSCLC cell viability in the presence of DDP resistance. Additionally, in our study, we provide another hypothesis that targeted inhibition of PTEN by miR-221 appears to be part of the mechanism by which TUG1 enhances chemotherapy sensitivity in NSCLC.

Investigations of TUG1 in lung cancer were previously reported. Several but not all studies were consistent with our hypothesis concerning low TUG1 expression level with NSCLC progression. These similar studies supported TUG1 lowly expressed in NSCLC, while they did not further investigate chemotherapy sensitivity [13, 19]. Contradictory to our results, Niu *et al.* showed that TUG1 knockdown promoted cell growth and induced chemoresistance of small-cell lung cancer (SCLC) [14]. However, a relatively small sample size (only thirty-three cases of SCLC recruited) may limit the power of statistical analysis, and absence of patient clinical response to chemotherapy may weaken the validity of results. In addition, in the study of Fang *et al.*, they explored the effect of TUG1 on xenografts derived from NSCLC patients, and none of these patients received chemotherapy [20], and the results showed that TUG1 downregulation inhibited the progression of patient-derived xenografts of NSCLC. However, only twenty-six NSCLC primary tumors yielded the establishment of nine patient-derived xenografts. A tumor take rate of 34.6% may limit the power of statistical analysis. Our study recruited 108 pathologically diagnosed NSCLC patients who received DDP-based chemotherapy and found that low expression levels of TUG1 may be associated with the resistance to DDP-based chemotherapy and poor overall survival. The TUG1 was originally identified as a transcript up-regulated by taurine [21]. Taurine possesses intrinsic anti-inflammatory and antioxidant abilities that may attenuate lipopolysaccharide-induced acute lung injury [22]. Likewise, TUG1 was found to protect against lipopolysaccharide-induced injury and it may be of benefit against inflammatory response and oxidation [23]. To the best of our knowledge, the role played by chronic inflammation in lung cancer has been proved [24, 25]. Therefore, we may speculate that TUG1 highlighted its anti-inflammatory role in NSCLC. In the future investigation, we may focus on the TUG1 and chronic inflammation in NSCLC progression after chemotherapy.

The molecular mechanisms by which TUG1 mediates NSCLC cells were various, including the p53/TUG1/ PRC2/HOXB7 interaction and the TUG1/CELF1/PRC2 axis [13, 19]. In the present study, we found TUG1 overexpression reduced miR-221 that targeted PTEN, suggesting TUG1/miR-221/PTEN as a mode in regulating chemotherapy sensitivity NSCLC. We determined higher expression of miR-221 and lower expression of PTEN in tissue samples from non-responders to platinum-based chemotherapy compared with responders. miR-221, carrying the same sequence of miR-222, is as a oncogene that was investigated in the development and chemotherapy response of several human cancers [26]. Zhang *et al.* demonstrated TUG1 expression was found be to be upregulated by p53, known as a tumor suppressor, in NSCLC [19], suggesting the positive interaction between TUG1 and p53. Furthermore, Fornari with his team demonstrated an elevated transcriptional activation of pri-miR-221 but a reduced mature miR-221 expression as results of stronger p53 expressions [27]. In this study, we used a computer-based lncRNA-miRNA target detection program that showed TUG1 bind to miR-221, which was evidenced by decrease of luciferase activity in vectors with TUG1 in the presence of miR-221. However, whether there is a loop that sustains miR-221 aberrant expression in NSCLC are required to elucidate in future studies. PTEN as a target of miR-221 was well-documented and its targeted inhibition by miR-221 enhanced drug resistance in many types of human cancers [28, 29]. A previous study reported miR-221 inhibited caspase-3-mediated apoptosis, thus modulating Sorafenib resistance in hepatocellular carcinoma [30]. Consistent with our results, TUG1 inhibited cell proliferation, migration and invasion, but increased apoptosis and autophagy of DDP resistant NSCLC cells. PTEN is a lipid phosphatase that removes phosphate groups from key intracellular phosphoinositide signaling molecules. This activity usually modulates growth and survival signals by PI3K signaling pathway. The loss of PTEN function in tumor cells results in constitutive stimulation of PI3K signaling pathway, increasing cellular proliferation [31]. In NSCLC, aberrant activation of PTEN/PI3K/AKT/mTOR pathway constitutes one of the most relevant mechanisms of acquired drug resistance [32]. Finally, these in vivo findings were reproduced in vitro on xenografts of DDP resistant NSCLC cells in BALB/c mice following DDP treatment and we found the anti-tumor effect of TUG1. Additionally, we demonstrated overexpression of TUG1 facilitated the senescence of NSCLC cells, as evidenced by reduced expression of hTERT in the presence of TUG1 in SPC-A1/DDP and H520/DDP cells. As previously reported, TUG1 was found to promote lens epithelial cell apoptosis by regulating miR-421/caspase-3 axis in age-related cataract [33]. We found TUG1 could bind to miR-221 which was known to inhibit the tumor suppressor PTEN. PTEN deactivates the PI3K/AKT/ hTERT pathway, thus promoting the senescence of lung adenocarcinoma cells [34].

In conclusion, we performed *in vitro* and *in vivo* studies that both verify the hypothesis that TUG1 overexpression is of benefit against chemotherapy resistance in NSCLC. TUG1 allows an increased PTEN by diminishing miR-221, which serves as a mode that mediates NSCLC cell response to chemotherapy. However, the mechanisms identified here deserve attention due to lack of specific mechanism between TUG1 and miR-221. Thus, it is also recommended in future studies to find out whether there is a feedback between TUG1 and miR-221.

## MATERIALS AND METHODS

### Ethics statement

This study was performed with the approval of the Ethics Committee in The fourth hospital of Hebei Medical University. Informed consent and required documentation was obtained from each patient and respective guardians prior to the study. All animal procedures were conducted in accordance with the National Institutes of Health Guide for the Care and Use of Laboratory Animals (National Institutes of Health, Bethesda, MA, USA).

### Patients

This study recruited 108 pathologically diagnosed NSCLC patients (64 males and 44 females, mean age = 56.52 ± 12.57 years) who received surgical resection from March, 2015 to March, 2016. According to the 7^th^ edition of TNM staging system proposed by the International Association for the Study of Lung Cancer (IASLC) [35], 42 patients were determined as stage I and 66 patients with stage II-IV. Besides, there were 50 cases of adenocarcinoma and 58 cases of squamous cell carcinoma. Patients were included when: 1, they were primary NSCLC patients, excluding recurrence cases; 2, they did not receive radiotherapy, chemotherapy or other neoadjuvant treatment before surgery; 3, their diagnoses were confirmed by professional pathologists according to the NSCLC histopathological diagnostic criteria; 4, no history of other malignant tumors; 5, they have complete clinical pathological and postoperative follow-up data. The chemotherapy regimen was described as follows. 108 patients received cisplatin (DDP)-based chemotherapy: first with first-line chemotherapy (etoposide + DDP) chemotherapy and second-line chemotherapy (irinotecan + DDP) after drug resistance. Among them, 43 patients were sensitive to chemotherapy (the tumor was reduced by more than 30% or disappeared after 4 to 5 cycles of chemotherapy) and 65 patients were resistant to chemotherapy (the tumor increased by more than 30% after 4 to 5 cycles of chemotherapy, or new metastatic lesions occurred). These 65 patients with drug resistance were treated with second-line chemotherapy. All patients were followed up after discharge, mainly by telephone and clinic visits. The follow-up included general conditions, clinical symptoms and imaging examinations (thoracic and abdominal CT, ultrasound examination of superficial lymph nodes). The follow-up was started from the date of surgery or pathological biopsy to death or the date of March 31, 2018. By the end of follow-up, 36 patients survived and 72 died. The Kaplan-Meier method was used to plot the survival curves of the patients.

### Cell culture

NSCLC cell lines SPC-A1, NCI-H520, NCI-H520, NCI-H1299 and normal lung epithelial cell line 16HBE were purchased from the Cell Bank of the Chinese Academy of Sciences (Shanghai, China). The lung cancer cell line SPC-A1 were cultured in high-glucose DMEM and the cell lines NCI-H1650, NCI-H520 and NCI-H1299 in RPMI-1640 medium (Gibco, Grand Island, NY, USA). The 16HBE cells were cultured by high-glucose DMEM containing 10% FBS, 100 U/ml penicillin, and 100 mg/L streptomycin in a constant temperature incubator containing 5% CO2 at 37°C. Fresh medium was replaced every 1-2 days. The cells were passaged when the cell confluency reached 80%–90%. After comparing the difference in expression of TUG1 between lung cancer cell lines and 16HBE cells, we finally selected SPC-A1 and NCI-H520 cells for subsequent experiments. The drug-resistant cell lines SPC-A1/DDP and NCI-H520/ DDP were induced. Cells were conventionally cultured, and then cultured in RPMI-1640 medium containing DDP (0.05 μg/ml) at a low concentration when 50% of them adhered to the wall. After 24 hours, the cells were further cultured by fresh RPMI-1640 medium. The cells were passaged when they grew to confluence. The concentration of DDP (1 μg/ml) was gradually increased after passaging, until cells maintained the drug resistance after the cells are cultured in the drug-free medium for one month or after post-thaw recovery. The DDP-containing culture need to be stopped for at least 2 weeks before the experiment can be started.

### Cell treatment

For the TUG1 overexpression analysis, we synthesized human full-length TUG1 fragment, which was cloned into the pcDNA3.1 vector (Invitrogen, Carlsbad, CA, USA) and named pcDNA-TUG1. An empty pcDNA3.1 vector was used as a control. Three siRNAs specifically targeting TUG1 were designed and constructed [11]. A lentiviral vector (LV-shPTEN) was constructed using shRNA targeting human PTEN. A lentiviral vector carrying a non-specific sequence was used as a negative control. The lentivirus was packaged in 293T cells, and lentivirus cells were harvested and filtered 72 hours after transfection. Cells were infected in serum-containing medium supplemented with 8 μg/ml polybromo. After 48 hour of infection, cells were screened with 2 μg/ml puromycin (Sigma, St. Louis, MO, USA). Cells were pre-incubated in six-well plates till 60-80% confluence and plasmids or siRNAs were transfected with Lipofectamine 2000 (Invitrogen) and Opti-MEM (Gibco). MiR-221 mimic/negative control (NC) mimic and miR-221 inhibitor/NC inhibitor were purchased from Shanghai GenePharma Co., Ltd. (Shanghai, China).

### *in situ* hybridization

The cells were cultured on polylysine-treated coverslips, and the cells were washed three times with PBS. Then cells were fixed in 4% paraformaldehyde (containing 1/1000 DEPC, Sigma) at room temperature for 20-30 min, and washed with distilled water 3 times. Cells were treated with 30% H_2_O_2_ and pure methanol (mixed at a ratio of 1: 50) at room temperature for 30 min, and washed three times with distilled water. The 3% citric acid was added to digest freshly diluted pepsin for 5–120 s, followed by *in situ* hybridization and three PBS washes. Then cells were fixed in 1% paraformaldehyde (containing 1/1000 DEPC) at room temperature for 10 min and washed with distilled water for 3 times. 20 μl of pre-hybrid solution was added to each section, which was placed in a humidity chamber. The humidity chamber (Wuhan Boster Biological Technology Co., Ltd., Wuhan, Hubei, China) was incubated in an incubator at 38–42°C for 4 h. The excess liquid was aspirated, and 20 μl of the probe hybridization solution was added dropwise to each section, which was hybridized overnight at 38-42°C in an incubator. The sections were taken out and washed 1-2 times separately with 2 × SSC, 0.5 × SSC and 0.2 × SSC at about 37°C. The blocking solution was added dropwise, followed by incubation in a 37°C incubator for 30 min. Then, biotinylated rat anti-digoxigenin was added dropwise, followed by incubation for 2 h at 37°C and four PBS washes. 50 μl of streptavidin-biotin complex was added dropwise, followed by incubation at 37°C for 30 min and three PBS washes. 50 μl of biotinylated peroxidase was added dropwise, followed by incubation at 37°C for 30 min and three PBS washes. Color was developed by DAB for 5 min, and sections were counterstained with hematoxylin. After dehydration and transparency, the sections were sealed in glycerin and then observed under a confocal microscope (Olympus, Tokyo, Japan).

### Nuclear and cytoplasmic RNA segmentation

The cells in logarithmic growth phase were trypsinized. 10^7^ cells were collected, centrifuged at 72 × g for 5 min, followed by three washes with ice-cold PBS. With the PBS discarded, 500 μl of ice-cold cell fractionation buffer was added, gently shaken, and lysed on ice for 10 min. Centrifugation was performed at 500 × g for 5 min at 4°C to collect the supernatant (cytoplasm) in a new RNase-free EP tube. The deposition (nucleus) was washed with 500 μl ice-cold cell fractionation buffer, followed by centrifugation at 500 × g for 1 min at 4°C to discard the supernatant. An equal volume of cell disruption buffer was added to the supernatant (cytoplasm) to lyse the nuclear deposition. An equal volume of 2 × lysis/binding solution and 100% ethanol was added to the above nucleus and cytosolic lysate. The mixture was centrifuged at 10,000 × g for 1 min at 4°C. A RNA recovery kit (Sangon Biotech Co., Ltd., Beijing, China) was adopted to collect solution for quantitative real-time polymerase chain reaction (qRT-PCR) detection.

### RNA immunoprecipitation (RIP)

RIP analysis was performed using the Magna RIP RNA binding protein immunoprecipitation kit (Millipore, Billerica, MA, USA). Cell lysates were incubated with magnetic beads-containing RIP buffer in combination with human anti-Argonaute 2 (Ago2) antibody (Abcam, Cambridge, MA, USA), with the total RNA as an input control (Input) and IgG as a negative control. After the protein was digested with proteinase K, the immunoprecipitated RNA was isolated and purified, and subjected to qRT-PCR analysis.

### qRT-PCR

Total RNA was extracted from tissues and cells by the method of Trizol (Invitrogen), and reverse transcription was carried out according to the instructions of the PrimeScript RT reagent Kit (Takara, Japan) to obtain cDNA. mRNA was quantified by qRT-PCR using SYBR Premix Ex Taq II (Takara, Otsu, Shiga, Japan) as indicated in the instructions. The primers were synthesized by Genomics Institute (Shenzhen, Guangdong, China). U6 and GAPDH were used as internal references. The melting curve was used to evaluate the reliability of PCR results. The CT value (inflection point of amplification curve) was taken. ΔCt = CT (target gene) - CT (internal reference), ΔΔCt = ΔCt (experimental group) - ΔCt (control group), and the relative expression of the target gene was calculated by 2-ΔΔCt. The experiment was repeated three times, and mean value was obtained. Primer sequences were as follows: TUG1, Forward: 5′-TAGCAGTTCCCCAAT CCTTG-3′, Reverse: 5′-CACAAATTCCCATCATTCC C-3′; miR-221, Forward: 5′-CATGGTCCTGCTGGAG TTCGTG-3′, Reverse: 5′-ATTTGTAACCATTATAAG CTGC-3′; PTEN, Forward: 5′-CCGGAATTCACCATG ACAGCCATCAT-3′, Reverse: 5′-GCGTCGACTTATC AGACTTTTGTAATT-3′; U6, Forward: 5′-CGCTTCG GCAGCACATATACTA-3′, Reverse: 5′-CGCTTCAC GAATTTGCGTGTCA-3′; GAPDH, Forward: 5′-AGC CACATCGCTCAGACAC-3′, Reverse: 5′-GCCCAAT ACGACCAAATCC-3′.

### Dual luciferase reporter assay

293T cells were seeded at 4 × 10^4^ cells/well in 24-well plates, and the cells were co-transfected with pmir-GLO/pmirGLO-TUG1-WT/MUT reporter plasmid and miR-221 mimic or miR-NC on the next day. The pmir-GLO/pmirGLO-PTEN-WT/MUT reporter plasmid was also co-transfected with miR-221 mimic or miR-NC. 24 hours after transfection, relative luciferase activity was determined using a dual luciferase reporter assay system (Promega, Madison, WI, USA) and Renilla luciferase activity was normalized.

### Fluorescence microscopic observation of GFP-LC3 fusion protein

The principle of aggregation when forming autophagy of LC3 was as follows: if autophagy does not occur, the GFP-LC3 fusion protein will be dispersed in the cytoplasm; after autophagy, the fusion protein will be transferred on the autophagosome membrane. At this time, a large number of green fluorescent spots can be found under the fluorescence inverted microscope, and each bright spot can be regarded as an autophagosome at this time. The autophagy activity can be evaluated by the number of fluorescent spots, and the autophagy activity is positively correlated with the number of fluorescent spots. The cells were seeded in a 6-well plate. When the cell confluency reached 50% - 60%, the GFP-LC3 plasmid (Plasmid #11546; Addgene, Cambridge, MA, USA) was transfected into cells by Lipofectamine 2000, based on instructions. The number of green fluorescent spots was calculated under the fluorescence microscopy using ImageJ software (National Institutes of Health, Bethesda, MA, USA).

### Monodansylcadaverine staining

MDC is a specific in vivo marker of autophagic vacuoles. SPC-1A/DDP and NCI-H520/DDP cells cultured on coverslips were treated with 5 μM 4-hydroxy tamoxifen (4-OHT) for 48 h, and then 50 μM MDC (Sigma-Aldrich Chemical Company, St Louis MO, USA) was used for staining for 15 min in the dark at 37°C. After being washed twice with PBS, the cells were observed under a fluorescence microscope (Leica, Wetzlar, Germany). The MDC-positive cells showed a blue-green or yellow-green granular structure at an excitation wavelength of 355 nm and an emission wavelength of 460 nm.

### 3-(4,5-dimethylthiazol-2-yl)-2,5-diphenyltetrazolium bromide (MTT)

The cells were trypsinized to prepare a single cell suspension, which was seeded in a 96-well plate at a density of 5 × 10^3^ cells per well. 200 μl of RPMI-1640 medium was added to each well. After the cells adhered to the well after 24 hours of culture, the culture medium was carefully aspirated out. The culture medium containing different concentrations of chemotherapeutic drugs was added to each well, and three duplicate wells were set for each concentration. After 48 hours of culture, 20 μl of MTT solution (5 mg/ml) was added to each well for further 5 hours of culture. The 96-well plate was taken out from the incubator, and the liquid in the plate was aspirated. Each well was mixed with 100 μl of DMSO and shaken for 10 min. The absorbance at a wavelength of 490 nm was measured in an enzyme-linked immunosorbent assay to calculate the IC50.

### Colony formation assay

After transfection for 48 h, the cells in logarithmic growth phase were inoculated into 6-well culture plates (200 cells/well), and 3 replicate wells were set up. The cells were cultured for 2 weeks, until the white colony spots were visible to the naked eye, when the culture was terminated. 2 mL of methanol was added to fix the plate for 15 min at room temperature. Then Giemsa staining solution was added to incubate for 15 min at room temperature, followed by slow rinsing and air drying. The number of cell colonies was observed and counted under a light microscope. The experiment was repeated three times.

### Flow cytometry

The cells were seeded in a 6-well culture plate with 1 × 10^6^ cells per well, and different concentrations of DDP (0, 10, 50 μmol/L) were added. The cells were transfected for 24 hours and then resuspended in PBS to prepare a single cell suspension. The cells were resuspended in pre-cooled 75% ethanol and fixed at 4°C overnight. The supernatant was discarded after centrifugation, followed by two washes with PBS. 450 μl PBS was used to resuspend the cells for each sample. Then 50 μL of propidium iodide (PI, 0.5 mg/ml) was added, followed by 37°C water bath for 30 min. The supernatant was discarded after centrifugation, followed by resuspending the cells in PBS. The cell cycle distribution was analyzed by flow cytometry (FACSCalibur, BD Biosciences, Franklin Lakes, NJ, USA). The experiment was repeated three times independently.

The corresponding cells were collected, and the cell density was adjusted to 5 × 10^5^. After this, the cells were seeded (2 ml/well) in 6-well plates, and different concentrations of DDP (0, 10, 50 μmol/L) were respectively inoculated. After 48 hours, the cells were collected and washed with PBS. Then, the plates were placed in 500 μl of pre-cooled 1 × binding buffer, and 5 μl Annexin-V-FITC and 2.5 μl PI were added in the cell suspension, followed by test on the flow cytometer (FACSArial I cell sorter, BD Biosciences). The results showed healthy viable cells in the lower left quadrant (Q4) on the scatter plot as (FITC^-^/PI^-^). The lower right quadrant (Q3) represented the early stage apoptotic cells as (FITC^+^/PI^-^). The upper right quadrant (Q2) represented necrotic cells and late stage apoptotic cells as (FITC^+^/PI^+^). Apoptotic rate = percentage of early stage apoptotic cells (Q3) + percentage of late stage apoptotic cells (Q2). The experiment was repeated three times independently.

### Scratch test

The cells after 48 h of transfection were inoculated into 5 well plates at a density of 5 × 10^5^ cells/well. When the cell confluency reached 80%, a straight line in the vertical direction was drawn crossing each well through the midline with a 1 ml sterile pipette tip. The exfoliated cells were rinsed off with PBS. With the medium added, the plates were incubated at 37°C in a 5% CO_2_ incubator for 24 h, followed by fixation with 75% ethanol at 4°C for 30 min. Then plates were subjected to hematoxylin-eosin staining and neutral gum sealing. Under the light microscope, the changes of the scratch width of any three slides in each group were observed. The cell migration distance was measured with mean value obtained.

### Transwell assay

40 μl of Matrigel dissolved at 4°C was added to the pre-cooled Transwell chamber, and incubated for 1 h in a cell culture incubator for gelatinizing. The cells after 48 h of transfection were adjusted to a cell concentration of 1 × 10^5^ cells/100 μl with serum-free medium. 100 μl of the cell suspension was added to the upper chamber of Transwell chamber (BD Biosciences) placed in a 24-well culture plate. 500 μl of culture medium containing 10% FBS was added to the lower chamber, which was incubated for 48 hours at 37°C in a 5% CO_2_ incubator. The chambers were taken out. The excessive cells in the upper chamber were wiped off with a cotton swab, fixed in 4% paraformaldehyde for 15 min, and washed once with PBS. The crystal violet was used for staining for 10 min, followed by one PBS wash. 5 fields were randomly selected under a high power microscope (Olympus) to calculate the number of cells penetrating through the pores.

### Western blot assay

Tissue or cells in logarithmic growth phase were added to the cell lysate to extract protein. The protein concentration was determined by BCA method (Sigma). The uploading volume of the sample was adjusted according to the protein quantification result. 30 μg was loaded per well. The protein was separated by SDS-PAGE and transferred onto the PVDF membrane. The membrane was blocked by 50 g/L skim milk powder for 1 h, followed by TBST solution washing for 3 times. Then the samples were incubated with primary antibodies rabbit anti-human PTEN (1:1000, Cat No. 32199, Abcam, Cambridge, UK), Beclin1 (1:1000, Cat No. 62557, Abcam), LC-3 (1:3000, Cat No. 51520, Abcam), hTERT (1:1000, Cat No. 32020, Abcam), and β-actin (1:5000, Cat No. 8227, Abcam) overnight at 4°C on a shaker. After three washes with TBST, HRP-labelled goat anti-rabbit IgG antibody (1:3000 dilution, Cat no. 205718, Abcam, Cambridge, UK) was added for incubation for 1 h, followed by TBST wash for 3 times. The target band was detected by ECL chemiluminescence and recorded using the Bio-image system (Bio-Rad, Hercules, CA, USA). The level of protein expression was normalized with β-actin as an internal reference.

### Cellular tumorigenicity in nude mice

Female BALB/c nude mice (4-6 weeks old) were purchased from Shanghai Slac Laboratory Animal Co. Ltd. (Shanghai, China) and randomly divided into 4 groups (5 in each group). 2 × 10^6^ SPC-A1/DDP (or NCI-H520/DDP) cells stably transfected with si-control or si-TUG1 were injected subcutaneously to establish a xenogenic transplantation model. The tumor growth was monitored and the tumor volume (V) was calculated, V = L × W^2^ × 0.52. L is the long diameter of the tumor, and W is the short diameter of the tumor. The growth curve of the transplanted tumor was drawn. The mice were sacrificed 4 weeks later and the tumors were weighed. The lung tissues of the mice were extracted and fixed with 4% paraformaldehyde overnight, followed by washing with double distilled water. Then the tissues were dehydrated, de-alcoholized, permeabilized by chloroform, embedded in paraffin, and then sliced continuously at a thickness of 3-4 μm for subsequent experiments.

### Terminal deoxynucleotidyl transferase-mediated dUTP nick-end labeling (TUNEL) assay

From the lung tissue sections of the mice, no less than 3 sections were selected. After dewaxing and hydrating, the Proteinase K working solution (20 μg/ml) was used to hydrolyze slices for 15-30 min. Following rinsing with PBS for 5 min × 4 times, 20% normal bovine serum was added to incubate slices for 30 min at room temperature. Two drops of TdT buffer were added dropwise to incubate for 5-10 min. The TUNEL reaction mixture (Roche, Alameda, CA, USA) was added and the slices were preserved on the ice for use as indicated by the instructions. 50 μl of TdT reaction solution was added dropwise for the reaction in a humidity chamber for 1 h, which was then incubated for 30 min in the reaction stopping buffer preheated to 37°C. After rinsing in PBS for 5 min × 4 times, the freshly prepared 0.05% DAB was added dropwise to develop color at 37°C for 10 min. After rinsing in PBS for 5 min × 4 times, the slices were subjected to counterstaining, washing, permeabilizing, and sealing. The positive expression was represented by brownish yellow. Under the optical microscope (400 ×), 10 non-overlapping fields were randomly selected and photographed, and the total number of TUNEL positive cells was recorded.

### Statistical analysis

The data were analyzed by SPSS 21.0 (IBM Corp, Armonk, NY, USA) statistical software. The statistical data with normal distribution were compared by Chi-square test. The measurement data were expressed by mean ± standard deviation. Pairwise comparisons were performed using the least significant difference (LSD) method. Comparisons among multiple groups were performed using one-way ANOVA. The comparison of data with normal distribution between two groups was analyzed using *t* test. Correlation analysis was performed using Pearson correlation analysis. Statistical significance was defined at a level of 5% (*p* < 0.05).
